# *In vivo* PET Imaging of Gliogenesis After Cerebral Ischemia in Rats

**DOI:** 10.3389/fnins.2020.00793

**Published:** 2020-07-30

**Authors:** María Ardaya, Ana Joya, Daniel Padro, Sandra Plaza-García, Vanessa Gómez-Vallejo, Mercedes Sánchez, Maider Garbizu, Unai Cossío, Carlos Matute, Fabio Cavaliere, Jordi Llop, Abraham Martín

**Affiliations:** ^1^Achucarro Basque Center for Neuroscience, Leioa, Spain; ^2^Department of Neuroscience, University of Basque Country (UPV/EHU) and CIBERNED, Leioa, Spain; ^3^CIC biomaGUNE, Basque Research and Technology Alliance, San Sebastian, Spain; ^4^Centro de Investigación Biomédica en Red – Enfermedades Respiratorias, CIBERES, Madrid, Spain; ^5^Ikerbasque Basque Foundation for Science, Bilbao, Spain

**Keywords:** Cerebral ischemia, PET, [^18^F]FLT, gliogenesis, glia

## Abstract

*In vivo* positron emission tomography of neuroinflammation has mainly focused on the evaluation of glial cell activation using radiolabeled ligands. However, the non-invasive imaging of neuroinflammatory cell proliferation has been scarcely evaluated so far. *In vivo* and *ex vivo* assessment of gliogenesis after transient middle cerebral artery occlusion (MCAO) in rats was carried out using PET imaging with the marker of cell proliferation 3′-Deoxy-3′-[18F] fluorothymidine ([^18^F]FLT), magnetic resonance imaging (MRI) and fluorescence immunohistochemistry. MRI-T_2_W studies showed the presence of the brain infarction at 24 h after MCAO affecting cerebral cortex and striatum. *In vivo* PET imaging showed a significant increase in [^18^F]FLT uptake in the ischemic territory at day 7 followed by a progressive decline from day 14 to day 28 after ischemia onset. In addition, immunohistochemistry studies using Ki67, CD11b, and GFAP to evaluate proliferation of microglia and astrocytes confirmed the PET findings showing the increase of glial proliferation at day 7 after ischemia followed by decrease later on. Hence, these results show that [^18^F]FLT provides accurate quantitative information on the time course of glial proliferation in experimental stroke. Finally, this novel brain imaging method might guide on the imaging evaluation of the role of gliogenesis after stroke.

## Introduction

Gliogenesis is the process by which glial progenitor cells differentiate into mature glia during development, and in the adult brain to maintain and regulate brain function (Lee et al., 2000). Following stroke, neurovascular disturbances lead to a massive neuroinflammatory reaction through the activation of glial cells such as microglia and astrocytes that amplifies ischemic damage and neuronal death (Moskowitz et al., 2010). In turn, proliferation of glial cells underlies compensatory mechanisms that likely contributes to neuronal restoration after brain injury (Rusznak et al., 2016).

*In vivo* positron emission tomography (PET) of neuroinflammation has been mainly focused on the evaluation of microglia/macrophage and astrocytic activation using radiolabeled ligands for the Translocator protein 18 kDa (TSPO) (Martin et al., 2010; Dupont et al., 2017; Pulagam et al., 2017). TSPO, a mitochondrial protein expressed in reactive glial cells, is considered the hallmark for neuroinflammation, as it is highly expressed after inflammatory reaction while low expression is shown in the healthy brain (Martin et al., 2010). For this reason, this biomarker has been widely used to monitor neuroinflammatory activation in major neurological diseases i.e., stroke, Alzheimer’s disease, Parkinson’s disease and multiple sclerosis, among others (Dupont et al., 2017). Despite the imaging of glial activation has been fully characterized with TSPO radiotracers during the last decade, the investigation of glial cell proliferation using PET imaging still remains unexplored. PET imaging of cellular proliferation has been mainly limited to the use of 3′-Deoxy-3′-[^18^F]fluorothymidine ([^18^F]FLT) in both preclinical and clinical routine (McHugh et al., 2018; Schelhaas et al., 2018; Bashir et al., 2020). [^18^F]FLT is an analog of thymidine which is phosphorylated by thymidine kinase-1 (TK-1), an enzyme expressed during the DNA synthesis and up-regulated during the S phase of the cell cycle (Shields et al., 1998). Once phosphorylated, [^18^F]FLT cannot be incorporated into the DNA and is metabolically trapped inside the cells. For this reason, the uptake and accumulation of [^18^F]FLT are used as surrogate of cellular proliferation (Cieslak et al., 2016). [^18^F]FLT has been extensively used in the field of oncology to characterize tumors and predict the response to personalized therapeutic approaches (Schelhaas et al., 2017). Therefore, the main application of this radiotracer in neurology has been restricted to the diagnosis of glioblastoma (Nikaki et al., 2017; Brahm et al., 2018). Nevertheless, few studies have reported the visualization of endogenous neural stem cells in living animals using [^18^F]FLT after focal cerebral ischemia, that could be used to monitor the effect of drugs aimed at expanding the neural stem cell niche (Rueger et al., 2010, 2012). Moreover, previous studies from our group and others showed that the lack of toll-like receptor 4 which is mainly expressed in microglia and astrocytes after brain ischemia, was able to increase neurogenesis using [^18^F]FLT-PET in the subventricular area after focal ischemia in mice (Caso et al., 2007, 2008; Moraga et al., 2016). Despite these findings, the use of [^18^F]FLT to monitor proliferation of glial cells has not been explored before. The purpose of the present study was to investigate non-invasively gliogenesis in the rat brain after cerebral ischemia using [^18^F]FLT-PET and immunohistochemistry. In particular, we were interested in clarifying the relationship of the [^18^F]FLT uptake and proliferative microglia/macrophages and astrocytes in a preclinical model of ischemic stroke in rats. Hence, these results provide valuable information about the use of a novel imaging methodology to follow up proliferation of glial cells *in vivo* after cerebral ischemia. This research might ultimately contribute to a better design of novel diagnostic and therapeutic strategies for neurologic diseases such as stroke.

## Materials and Methods

### Cerebral Ischemia and Experimental Set-Up

Eight-weeks old male Sprague-Dawley rats (*n* = 32; 307 ± 4.3 g body weight; Janvier, France) were used for both non-invasive imaging and immunohistochemical studies. Animal experimental protocols and relevant details regarding welfare were approved by the animal ethics committee of CIC biomaGUNE and were conducted in accordance with the ARRIVE guidelines and Directives of the European Union on animal ethics and welfare. Rats were anaesthetized with 2.5% isoflurane in 100% O_2_ and transient focal ischemia was produced by a 90 min intraluminal occlusion of the middle cerebral artery (MCAO) followed by reperfusion as described previously (Justicia et al., 2001). Ischemic rats were subjected to T_2_-weighted (T_2_W) MRI scans at 24 h after reperfusion to select rats presenting cortico-striatal infarction for inclusion in the PET studies. 16 rats were used to carry out [^18^F]FLT-PET imaging studies. Eight of them were only scanned before ischemic insult. A different set of rats (*n* = 8) was scanned at 7 days after ischemia; of these, five animals were re-scanned at days 14 and 28 after ischemia. Overall, five animals were subjected to the three PET imaging sessions. Not all animals were subjected to the complete PET imaging protocol due to technical issues with the PET scanner at day 14 (*n* = 5) after stroke. In addition, one animal died before last PET session at day 28 (*n* = 7) after ischemia. In all cases, [^18^F]FLT uptake in the brain was determined. In addition, additional 16 rats were used to perform immunohistochemical studies for proliferation of glial cells before (control) and at days 7, 14, and 28 after ischemia.

### Magnetic Resonance Imaging

T_2_W-MRI scans were used to include rats in the PET study and to evaluate the infarction volume. Scans were performed in rats anaesthetized with 4% isoflurane and maintained by 2–2.5% of isoflurane in a 30/70% mixture of O_2_/N_2_. Animals were placed into a rat holder compatible with the MRI acquisition system and maintained normothermia using a water-based heating blanket at 37°C. To ensure animal welfare, temperature and respiration rate were continuously monitored while they remain in the MRI magnet, using a SAII M1030 system (SA Instruments, NY, United States). MRI *in vivo* studies were performed on a 7T horizontal bore Bruker Biospec USR 70/30 MRI system (Bruker Biospin GmbH, Ettlingen, Germany), interfaced to an AVANCE III console, and with a BGA12-S imaging gradient insert (maximal gradient strength 400 mT/m, switchable within 80 μs). These measurements were performed with a 72 mm volumetric quadrature coil for excitation and a 20 mm rat brain surface coil for reception. The imaging session started with the acquisition of a scout scan, which was used to plan the whole study focusing on the region of interest. T2-W images were acquired with a Bruker’s RARE (Rapid Acquisition with Relaxation Enhancement) sequence (Effective TE = 40 ms, TR = 4400 ms, NA = 2; Matrix = 256 × 256 points; FOV = 25.6 mm × 25.6 mm; spatial resolution = 100 μm × 100 μm; 24 contiguous slices of 1 mm thickness covering the whole brain), which was used to quantify the volume of the lesion. For the image analysis, regions of interest (ROIs) were manually defined by the same researcher a blind fashion using the open source software 3D Slicer image analysis software (version 4.8^1^) for each rat on the region of increased signal in the ipsilateral hemisphere. The total lesion volume was calculated by summing the area of the infarcted regions of all slices affected by the lesion.

### Radiochemistry

The synthesis of 3′-Deoxy-3′-[^18^F]fluorothymidine ([^18^F]FLT) was performed as described earlier (Machulla et al., 2000), using a TRACERlab FXFN synthesis module (GE Healthcare). In brief, [^18^F]Fluoride was produced via the ^18^O(p,n)^18^F nuclear reaction in 2.9 ml of 98% ^18^O-enriched water. The [^18^F]F^–^ was trapped in a pre-conditioned QMA cartridge and transferred to the reactor by sequential elution with a solution of K_2_CO_3_ (3.5 mg) in water (0.5 ml) and a solution of Kryptofix K_2_._2_._2_ (15 mg) in acetonitrile (1 ml). After azeotropic evaporation, a solution containing the precursor (10 mg of 5′-O-Benzoyl-2,3′-anhydrothymidine in 1 ml of dimethylsulfoxide) was added and the fluorination reaction was allowed to occur at 100°C for 10 min. The reactor was then cooled at room temperature and 0.35 ml of 1% NaOH aqueous solution were added for the hydrolysis of protecting groups (5 min at 50°C). Finally, 0.75 ml of 0.2 M NaH_2_PO_4_ aqueous solution and 1.75 ml of mobile phase, consisting of 0.01 M NaH_2_PO_4_ solution (90%) and ethanol (10%), were added and the mixture was purified by HPLC, using a VP125/10 Nucleosil 100-7C18 semipreparative column (Macherey-Nagel) as stationary phase. The collected fraction (retention time = 13,14 min) was filtered through a 0.22 μm sterile filter to yield the final [^18^F]FLT solution. Average radiochemical yield was 7.5 ± 1.1% (EOS) in an overall production time of ca. 60 min. Radiochemical purity was higher than 95% in all cases.

### Positron Emission Tomography Scans and Data Acquisition

PET scans were performed using an eXplore Vista PET-CT camera (GE Healthcare, Waukesha, WI, United States). Scans were performed in rats anaesthetized with 4% isoflurane and maintained by 2–2.5% of isoflurane in 100% O_2_. Animals were placed into a rat holder compatible with the PET acquisition system and maintained normothermia using a water-based heating blanket at 37°C. To ensure animal welfare, temperature and respiration rate were continuously monitored while they remain in the PET scanner, using a SAII M1030 system (SA Instruments, NY, United States). The tail vein was catheterized with a 24-gage catheter for intravenous administration of the radiotracer. For longitudinal assessment of [^18^F]FLT uptake after ischemia, control animals (*n* = 8) and ischemic animals at days 7 (*n* = 8), 14 (*n* = 5), and 28 (*n* = 7) after ischemia were scanned. The radioactivity (±70 MBq) controlled for animal weight was injected and dynamic brain images were acquired for 18 frames and 60 min in the 400–700 keV energetic window. After each PET scan, CT acquisitions were also performed (140 mA intensity, 40 kV voltage), to provide anatomical information of each animal as well as the attenuation map for the later PET image reconstruction. Dynamic acquisitions were reconstructed (decay and CT-based attenuation corrected) with filtered back projection (FBP) using a Ramp filter with a cutoff frequency of 0.5 mm^–1^.

### Positron Emission Tomography Image Analysis

PET images were analyzed using PMOD image analysis software (version 3.5, PMOD Technologies Ltd., Zurich, Switzerland). To verify the anatomical location of the signal, PET images were co-registered to the anatomical data of a MRI rat brain template. Two type of Volumes of Interest (VOIs) were established as follows: (1) A first set of VOIs was defined to study the whole brain [^18^F]FLT PET signal. Whole brain VOIs were manually drawn in both the entire ipsilateral and contralateral hemispheres on slices of a MRI (T_2_W) rat brain template provided by PMOD software. (2) A second set of VOIs was automatically generated in the cerebral cortex and the striatum by using the regions proposed by the PMOD rat brain template, to study the evolution of [^18^F]FLT PET signal in these specific regions in both the ipsilateral and contralateral cerebral hemispheres. The last five frames (last 15 min of acquisition) were summed to quantify radiotracer uptake. Average values in each ROI were determined and expressed as percentage of injected dose per cubic centimeter (%ID/cc).

### Immunohistochemistry and Cell Counting

Immunohistochemistry staining was carried out at control (*n* = 4), day 7 (*n* = 4), day 14 (*n* = 4), and day 28 (*n* = 4) after reperfusion and the “*n*” of each group was randomly assigned. The brain was removed, frozen and cut in 5-μm-thick sections in a cryostat. Sections were fixed in 4% paraformaldehyde during 15 min, washed with phosphate-buffered saline (PBS) and incubated 5 min in NH_4_Cl, following by two PBS rinse and methanol-acetone (1:1) permeabilization during 5 min at −20°C. After PBS washing, samples were saturated with a solution of bovine serum albumin (BSA) 5%/Tween 0.5% in PBS during 15 min at room temperature, and incubated during 2 h at room temperature with primary antibodies BSA (5%)/Tween (0.5%) in PBS. Sections were stained for Ki67 with rabbit anti-rat Ki67 (1:400, AbCam, Cambridge, United Kingdom), for CD11b with mouse anti-rat CD11b (1:300; Serotec, Raleigh, NC, United States) and for the glial fibrillary acidic protein (GFAP) with chicken anti-rat GFAP (1:500; AbCam, Cambridge, United Kingdom). Sections were washed (3 × 10 min) in PBS and incubated for 1 h at room temperature with secondary antibodies Alexa Fluor 594 goat anti-rabbit IgG, Alexa Fluor 488 goat anti-mouse IgG and Alexa Fluor 647 goat anti-chicken IgG (Molecular Probes, Life Technologies, Madrid, Spain, 1:1,000) in BSA 5%/Tween 0.5% in PBS, washed again (3 × 10 min) in PBS, and mounted with a prolong antifade kit with DAPI in slices (Molecular Probes Life Technologies, Madrid). Standardized images acquisition was performed with the Panoramic MIDI II automated digital slide scanner (3DHistech Ltd., Hungary) and the Leica SP8 microscope (Hospitalet de Llobregat, Spain). Cells were manually counted in ten representative and different fields at Bregma: circa 0.50 mm and 40× magnification in a blind-fashion to avoid bias by using Image J (Version 2.0.0-rc-69/11.52p, NIH) software.

### Statistical Analyses

For PET, the percentage of injected dose per cubic centimeter (%ID/cc) within each region and time point following cerebral ischemia were averaged and compared with the averaged baseline control values (day 0) using one-way ANOVA followed by Dunnet’s multiple-comparison tests for *post hoc* analysis. Likewise, cellular expression of both Ki67^+^/CD11b^+^ and Ki67^+^/GFAP^+^ at control and days 7, 14, and 28 after ischemia were compared using the same statistical analysis than that for PET imaging. The level of significance was regularly set at *p* < 0.05. Statistical analyses were performed with GraphPad Prism version 8 software.

## Results

**FIGURE 1:**
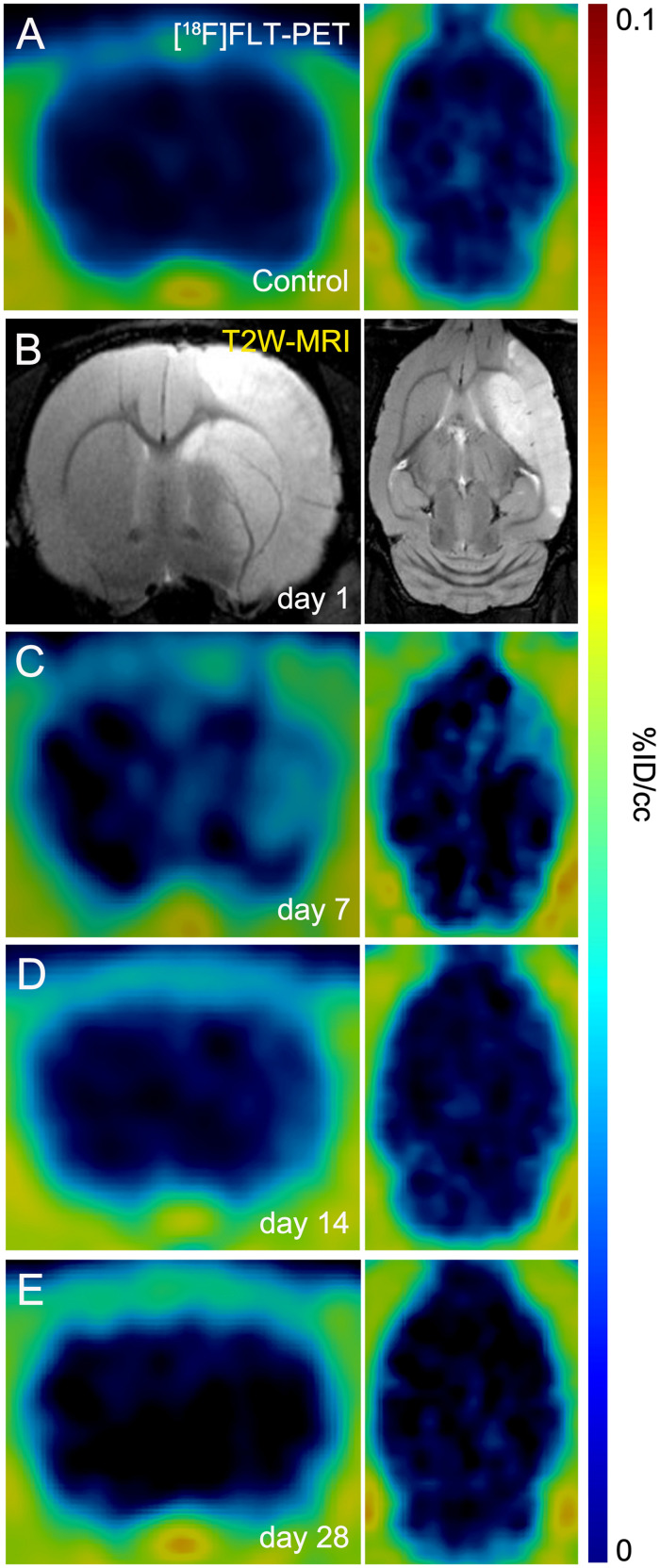
Positron emission tomography (PET) images of [^18^F]FLT and Magnetic resonance imaging (MRI) [T_2_-weighthing (T_2_W)] before (control) and at days 1, 7, 14, and 28 after cerebral ischemia in rats. [^18^F]FLT PET **(A, C–E)** and MRI-T_2_W **(B)** images of axial and coronal planes at the level of the lesion are shown. Images correspond to representative animals for each time point.

The evaluation of *in vivo* gliogenesis was explored by PET using the radiotracer [^18^F]FLT during the first month after transient focal ischemia in rats. All the images were quantified in standard units, i.e.,%ID/cc of [^18^F]FLT. The images with normalized color scale illustrate the evolution of the PET signals before (control) and at 7, 14, and 28 days after ischemia onset (Figures 1A,C–E). Hyperintensity of MRI-T_2_W images showed the edema formation as result of the evolution of the brain infarction at day 1 after reperfusion (Figure 1B). All animals subjected to PET studies showed cortico-striatal MRI alterations (mean ± sd: 298 ± 71 mm^3^, *n* = 8).

### Imaging Glial Proliferation Using [^18^F]FLT PET After Cerebral Ischemia

**FIGURE 2:**
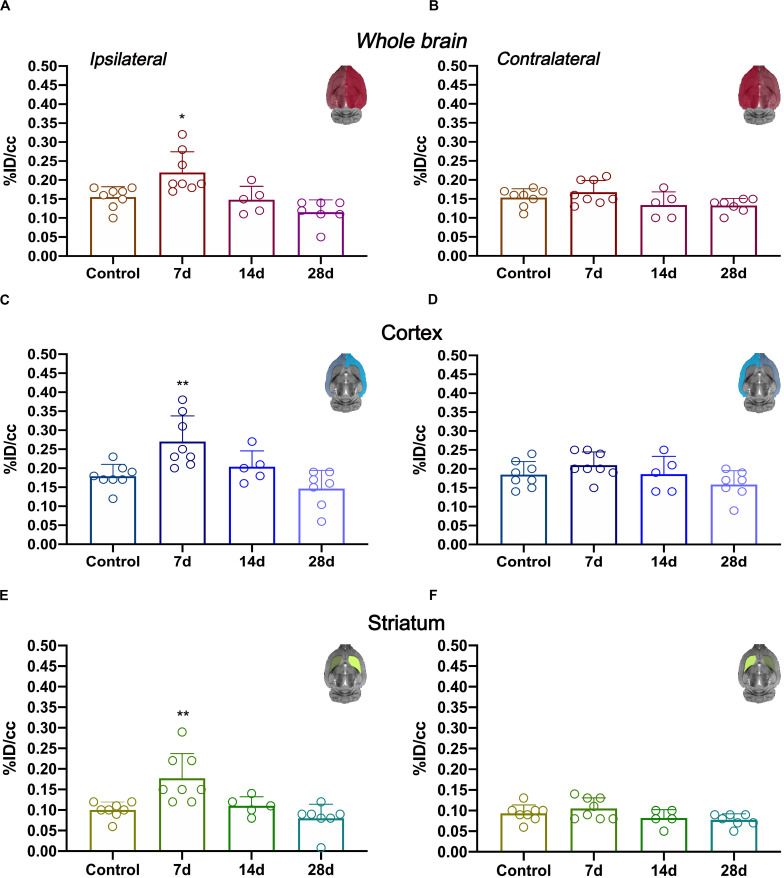
Time course of the progression of [^18^F]FLT PET signals at before (control) and after cerebral ischemia.%ID/cc (mean ± SD) of [^18^F]FLT was quantified in six VOIs. The entire ipsilateral cerebral hemisphere **(A)**, contralateral hemisphere **(B)**, ipsilateral cerebral cortex **(C)**, contralateral cerebral cortex **(D)**, ipsilateral striatum **(E)** and contralateral striatum **(F)** are shown. The upper right panels of each figure show the selected brain ROIs for the quantification defined on a slice of a MRI (T_2_W) template. Rats were examined by PET at control (*n* = 8) and at 7 (*n* = 8), 14 (*n* = 5), and 28 (*n* = 7) days after ischemia. *p < 0.05, **p < 0.01 compared with control.

*In vivo* imaging of glial proliferation was evaluated with the uptake of [^18^F]FLT in the ipsilateral and contralateral whole brain, cerebral cortex and striatum before (control) and at 7, 14, and 28 days after MCAO (Figure 2). All brain regions evaluated in both brain hemispheres showed a similar PET signal evolution after ischemia. In the ipsilateral whole brain, [^18^F]FLT uptake showed a significant increase at day 7 after ischemia in comparison to baseline (*p* < 0.05, Figure 2A). Subsequently, the PET signal displayed a decline during days 14 and 28 after stroke. In the contralateral whole brain, [^18^F]FLT signal showed similar values along the different days evaluated (Figure 2B). The ischemic cerebral cortex showed a significant PET signal increase at day 7 after ischemia in comparison to control rats followed a progressive decrease from days 14 to 28 after MCAO (*p* < 0.001, Figure 2C). The contralateral cerebral cortex showed similar values at different days after ischemia in relation to control values (Figure 2D). Moreover, cerebral cortex showed higher PET signal values (0.15–0.20%ID/cc) in relation to striatum (circa 0.10% ID/cc) in control rats, probably due to spill-over effects from the [^18^F]FLT PET signal coming from boundary tissues outside the brain. The injured striatum showed [^18^F]FLT PET signal uptake during the first week after reperfusion followed by a sharp decrease at the third and fourth week after ischemia (*p* < 0.001 with respect to control animals, Figure 2E). Finally, the contralateral striatum displayed non-significant PET signal changes at different days after MCAO (Figure 2F).

### Brain Lesion Evolution After Ischemia

**FIGURE 3:**
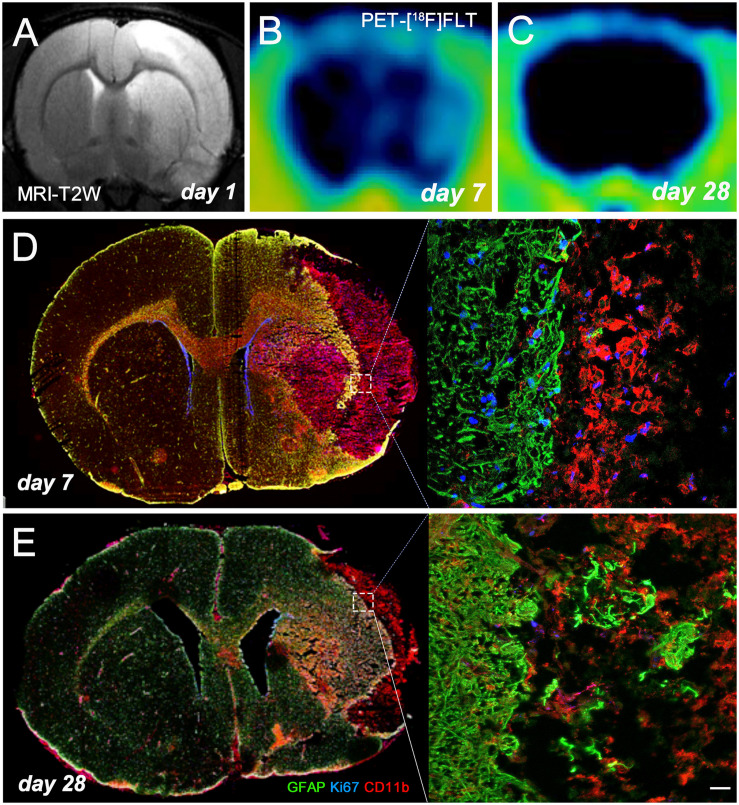
MRI-T_2_W and [^18^F]FLT PET signal shows the vasogenic edema and the evolution of cerebral proliferation at days 1 **(A)**, 7 **(B)**, and 28 **(C)** after MCAO. Images correspond to the same animal. Immunofluorescent labeling of GFAP (green), Ki67 (blue) and CD11b (red) of an axial section at days 7 **(D)** and 28 **(E)** shows gliogenesis after cerebral ischemia. Scale bar, 20 μm.

MRI-T_2_W image hyperintensities showed the formation of vasogenic edema as the consequence of brain infarction evolution at day 1 after ischemia onset (Figure 3A). Cortical and striatal MRI alterations coincided with the increase of [^18^F]FLT PET as potential marker for gliogenesis and the activation of microglia and infiltrated macrophages at day 7 (Figure 3B) followed by a decrease at day 28 (Figure 3C) after ischemia. The infarcted area (cerebral cortex and striatum) was occupied by cells with the morphology of amoeboid reactive microglia/macrophages showing intense CD11b immunoreactivity flanked by reactive astrocytes (GFAP^+^ cells) at day 7 after MCAO (Figure 3D). Some of these glial cells were positive for the proliferation marker Ki67, supporting the detection of glial proliferation by PET using [^18^F]FLT. During following weeks, brain infarction evolved into a cystic cavity surrounded by the formation of the astrocytic scar (Figure 3E).

### *Ex vivo* Evaluation of Glial Proliferation After MCAO

**FIGURE 4:**
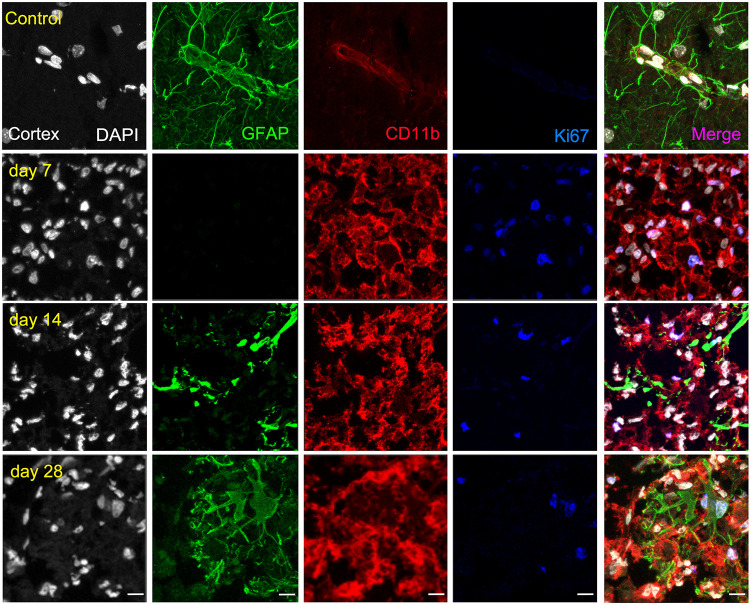
Immunofluorescent labeling of DAPI (white), GFAP (green), CD11b (red) and Ki67 (blue) in the ischemic cortex, shown as four channels. The data show temporal evolution of cortical proliferative (Ki^+^) microglia/macrophages (CD11b^+^) and astrocytes (GFAP^+^) at day 0 (control; first row), day 7 (second row), day 14 (third row), and day 28 (fourth row) after ischemia. Scale bars, 10 μm.

The ischemic cerebral cortex showed a significant increase of proliferative microglia/infiltrated macrophages (in red-CD11b and blue-Ki67; Figure 4) at day 7 in comparison to control brains followed by a slight decrease at day 14 and a sharp decline at day 28 after ischemia (*p* < 0.001, Figure 6A), In contrast, the number of proliferative astrocytes (in green-GFAP and blue-Ki67; Figure 4) showed pseudo-control values at day 7 followed by a mild non-significant increase at 14 and 28 days after cerebral ischemia (Figure 6A). Despite the number of CD11^+^/Ki67^+^ was much higher than GFAP^+^/ki67^+^cells at days 7 and 14, the values of both proliferative glial cells were similar at day 28 after MCAO (Figure 6A). In the non-ischemic cerebral cortex (contralateral), both proliferative microglia and astrocytes showed low values at different days after ischemia (Figure 6B).

**FIGURE 5:**
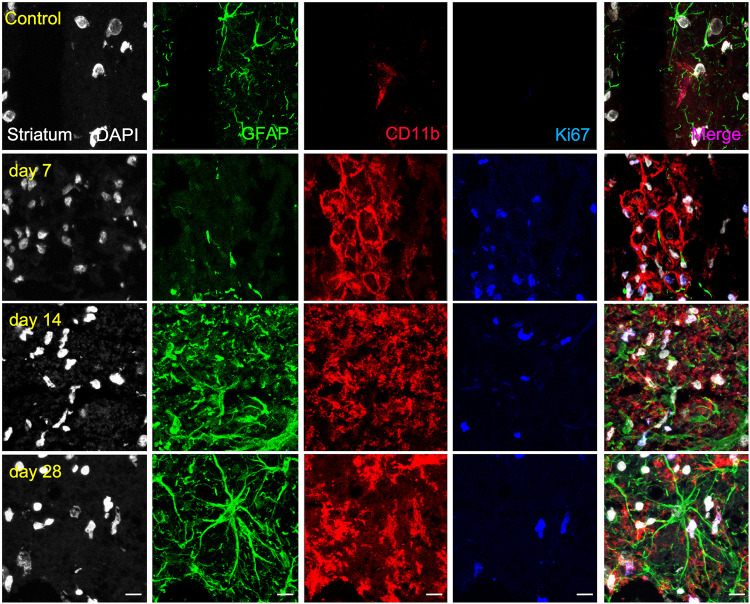
Immunofluorescent labeling of DAPI (white), GFAP (green), CD11b (red) and Ki67 (blue) in the ischemic striatum, shown as four channels. The data show temporal evolution of striatal proliferative (Ki^+^) microglia/macrophages (CD11b^+^) and astrocytes (GFAP^+^) at day 0 (control; first row), day 7 (second row), day 14 (third row), and day 28 (fourth row) after ischemia. Scale bars, 10 μm.

**FIGURE 6:**
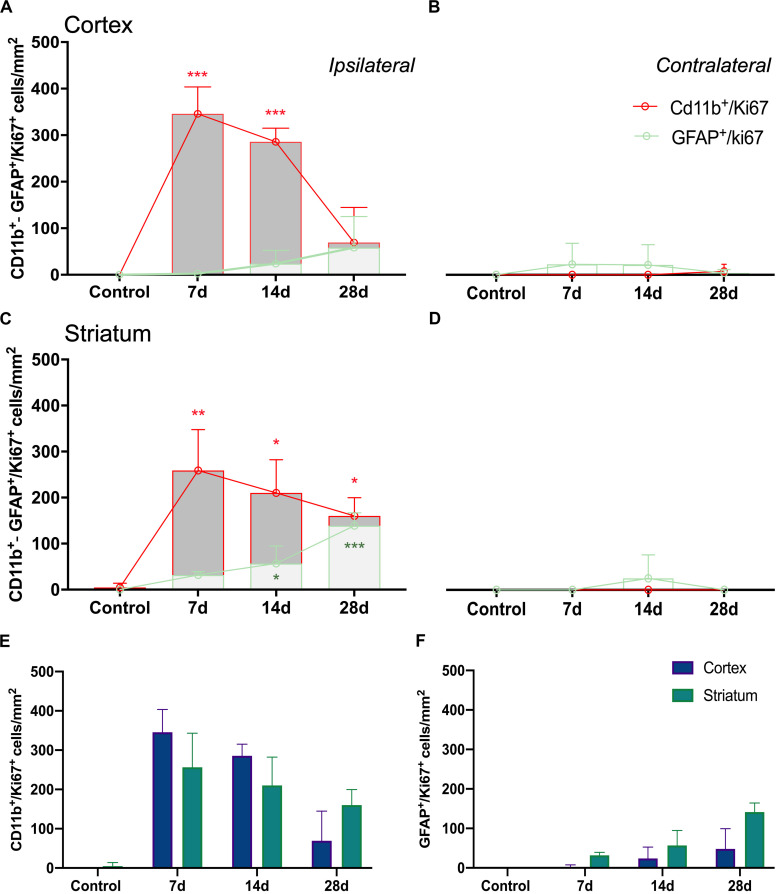
Temporal profiles of CD11b^+^/Ki67^+^ and GFAP^+^/Ki67^+^ cells in four brain regions after ischemia onset. Ipsilateral cerebral cortex **(A)**, contralateral cerebral cortex **(B)**, ipsilateral striatum **(C)** and contralateral striatum **(D)** are shown. CD11b^+^/Ki67^+^ **(E)** and GFAP^+^/Ki67^+^ **(F)** cells show evolution of proliferative glial cells in cerebral cortex and striatum. Cell counts/mm^2^ were counted before (control) and days 7, 14, and 28 (*n* = 12, 4 rats/time point) after ischemia. *p < 0.05, **p < 0.01, ***p < 0.001 compared with control.

Similarly, the ischemic striatum showed a significant increase of CD11^+^/Ki67^+^ (in red and blue; Figure 5) followed by a progressive decrease from days 14 to 28 after cerebral ischemia (*p* < 0.05; *p* < 0.01, Figure 6C). Likewise, the number of proliferative astrocytes showed a progressive mild increase from days 7 to 14 followed by the highest value reached at day 28 after MCAO (*p* < 0.05; *p* < 0.001, Figure 6C). In contrast, very low proliferative glial was observed in the contralateral striatum at the different time points after ischemia (Figure 6D). Cerebral cortex showed non-significant higher values of proliferative microglia and infiltrated macrophages at days 7 and 14 in relation to the striatum, while this situation was reverted at day 28 (Figure 6E). In the other hand, striatum showed non-significant increase of proliferative astrocytes in comparison to cerebral cortex at different days after stroke (Figure 6F).

## Discussion

The neuroinflammatory reaction, firstly led by the activation of microglia and the infiltration of macrophages and later by the over-reactivity of astrocytes, has been extensively monitored with PET after ischemic stroke (Rojas et al., 2007; Martin et al., 2010). However, new functions and properties of proliferative glia that might account for neuronal restoration (Rusznak et al., 2016) after brain ischemia have been barely studied so far. To fill this gap, we explored a novel non-invasive *in vivo* imaging method able to detect proliferation of microglia/macrophages and astrocytes after stroke in rats by using [^18^F]FLT PET.

Previously, Rueger and colleagues described the use of [^18^F]FLT PET as imaging method of endogenous neural stem cells (NSCs) after cerebral ischemia in rats (Rueger et al., 2010). This study showed for the first time the uptake of [^18^F]FLT in both NSC niches, the subventricular area and the hippocampus that correlated with a high bromodeoxyuridine (BrdU) accumulation in those brain regions. Additionally, these authors also described a cellular proliferation enhancement within the infarcted region with both [^18^F]FLT and BrdU staining at 1 week after preclinical stroke. However, these results were not directly related to the proliferation of glial cells rather than a merely increase of cellular proliferation after stroke (Rueger et al., 2010). Therefore, these latter findings are fully consistent with PET imaging results observed in our work (Figure 1). In the present study, the PET [^18^F]FLT signal uptake showed a significant increase at day 7 after ischemia that was followed by a progressive decline to pseudo-control values at day 28 after reperfusion (Figure 2). In fact, these results stand in agreement with the *in vivo* PET imaging distribution of TSPO, a well-known marker of neuroinflammation, after cerebral ischemia (Domercq et al., 2016). Hence, taking into account that the TSPO overexpression is principally due to the activation of microglia/infiltrated macrophages and astrocytes (Martin et al., 2010), our results could suggest that [^18^F]FLT signal in the region of infarction can be linked to the inflammatory reaction. To verify this hypothesis, we performed the immunohistochemical characterization of proliferative microglia/macrophages and astrocytes at days 7, 14, and 28 after ischemia (Figures 3–5).

In the present study, we evaluated the proliferative glial cells using the marker Ki67, a nuclear protein expressed in all phases of the cell cycle except the resting state. A previous study showed higher numbers of Ki67 positive cells in comparison to BrdU expression pattern in the rat brain. In fact, these findings might be due to BrdU is incorporated to DNA during the S-phase of the mitotic process, whereas Ki67 might be incorporated during all phases (Kee et al., 2002). Additionally, [^18^F]FLT has shown to be a thymidine analog that is trapped inside proliferative cells during the S-phase in a similar manner than BrdU. However, previous works showed lower [^18^F]FLT values in comparison to BrdU positive cells based on differences in their incubation times (i.e., from 1.5 to 6 h, respectively) (Rueger et al., 2010, 2012). Therefore, all these previous findings may explain the higher events observed in our study by immunohistochemistry with Ki67 positive cells in comparison to the PET signal with [^18^F]FLT (Figures 4–6). Despite this, these results showed a significant increase of proliferative microglia/macrophages at day 7 followed by a decrease from days 14 to 28 supporting the findings observed with [^18^F]FLT. In contrast, the number of proliferative astrocytes showed low levels during the first two weeks that was significantly increased at day 28 after ischemia. The increase of proliferative astroglial cells at day 28 was not paralleled by increased [^18^F]FLT uptake, which displayed pseudo-control values in both cerebral cortex and striatum one month after ischemia. In fact, these differences could be explained by the higher resolution showed by confocal microscopy in relation to PET imaging which allows for a more accurate detection of proliferative cells in the region of infarction (Tichauer et al., 2015).

Overall, our study showed that [^18^F]FLT PET was able to detect mainly proliferative CD11b positive cells (microglia and infiltrated macrophages) that accounted for the highest proliferative cellular population in the ischemic area at day 7 after stroke.

### Summary and Conclusion

In summary, we report here for the first time a novel imaging tool to evaluate glial proliferation using PET imaging. We describe a novel imaging tool to evaluate glial proliferation with PET since [^18^F]FLT is a radiotracer able to detect cellular proliferation *in vivo*. Following cerebral ischemia, a massive amount of glial cells become activated and others proliferate too. Since neuronal population dies in the region of the infarction after stroke, we might be able to monitor proliferative glial cells involved in the immune response. Therefore, these results provide valuable information regarding the *in vivo* detection of proliferative glia after stroke that might contribute to the discovery of novel diagnostic tools for stroke care.

### Limitation Section

The main limitations of the present study are as follows: (i) the resolution of current PET cameras might certainly limit the use of this imaging technique for the evaluation of low proliferative glial density, (ii) the no correction to compensate for the effect of the surface coil in MRI studies, (iii) the regions of interest of MRI images were drawn by less than two researchers, and (iv) the count of cells was carried out manually.

## Data Availability Statement

The raw data supporting the conclusions of this article will be made available by the authors, without undue reservation.

## Ethics Statement

The animal study was reviewed and approved by Ethics committee of CIC biomaGUNE.

## Author Contributions

MA, AJ, DP, SP-G, VG-V, MS, MG, and UC performed the experiments and acquired the data. FC, CM, JL, and AM designed the experiments and analyzed the data, prepared the manuscript, and approved the final version of the manuscript. All authors contributed to the article and approved the submitted version.

## Conflict of Interest

The authors declare that the research was conducted in the absence of any commercial or financial relationships that could be construed as a potential conflict of interest.

## Footnotes

1 http://www.slicer.org
